# Advancements in Trauma-Induced Acute Kidney Injury: Diagnostic and Therapeutic Innovations

**DOI:** 10.3390/life14081005

**Published:** 2024-08-13

**Authors:** Sergio Lassola, Francesco Cundari, Giuseppe Marini, Francesco Corradi, Silvia De Rosa

**Affiliations:** 1 Department of Anesthesia and Intensive Care, Santa Chiara Hospital, 38122 Trento, Italy; sergio.lassola@apss.tn.it (S.L.); giuseppe.marini@apss.tn.it (G.M.); 2 Department of Surgical, Medical, Molecular Pathology and Critical Care Medicine, University of Pisa, 56126 Pisa, Italy; f.cundari@studenti.unipi.it (F.C.); francesco.corradi@unipi.it (F.C.); 3 Centre for Medical Sciences—CISMed, University of Trento, Via S. Maria Maddalena 1, 38122 Trento, Italy

**Keywords:** acute kidney injury, diagnostic precision, kidney ultrasound, strategic interventions, protocols, trauma-associated nephropathy

## Abstract

Acute kidney injury following trauma impacts patient recovery critically, necessitating an integrated approach to emergency care and nephrology. This review aims to provide a comprehensive understanding of trauma-induced nephropathy, highlighting recent advancements in pathophysiological insights, diagnostic techniques, and strategic interventions. Our key findings emphasize the role of biomarkers, like Neutrophil Gelatinase-Associated Lipocalin and Liver Fatty Acid-Binding Protein, and imaging techniques, such as contrast-enhanced ultrasound, in early AKI detection. Preventive strategies, including aggressive fluid resuscitation, avoidance of nephrotoxic agents, and hemodynamic optimization, are essential for mitigating AKI progression. Integrating these approaches into trauma care frameworks aims to enhance patient outcomes and set a foundation for future research and clinical improvements.

## 1. Introduction

**Figure 1:**
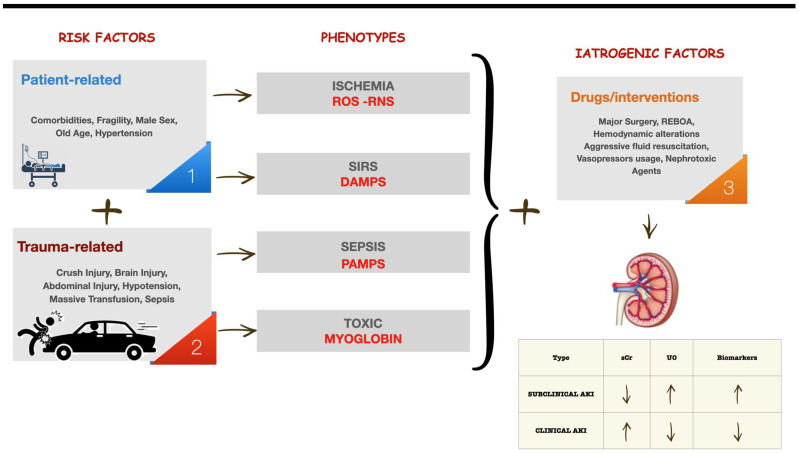
Risk factors, phenotypes, and iatrogenic factors leading to renal hypoperfusion and AKI in trauma patients.

Acute kidney injury (AKI) following trauma significantly impacts patient recovery, creating a crucial intersection between emergency care and nephrology [1]. Understanding trauma-induced nephropathy is essential for revealing the pathophysiological mechanisms and triggers of AKI. Trauma-induced AKI primarily results from direct physical damage to the kidneys, such as blunt or penetrating trauma, and systemic inflammatory responses triggered by such injuries. Mechanisms like rhabdomyolysis, where muscle breakdown products, particularly myoglobin, cause renal damage, and systemic inflammatory response syndrome (SIRS), which leads to widespread inflammation affecting renal perfusion, are central to this process [2] (Figure 1).

Conversely, post-ischemic kidney injury in organ transplantation is primarily caused by ischemia–reperfusion injury. This process involves temporarily depriving blood flow to the transplanted kidney (ischemia) and restoring circulation (reperfusion). The subsequent reoxygenation produces reactive oxygen species (ROS) and an inflammatory response that causes cellular damage and dysfunction [3]. While both trauma-induced AKI and post-ischemic kidney injury involve inflammatory responses, the triggers and specific pathways differ significantly. Trauma-induced AKI is directly related to physical injury and its systemic effects, whereas post-ischemic injury in transplantation is a result of surgical and perfusion-related factors [4,5]. Recent advancements in diagnostic approaches, including the application of biomarkers like Neutrophil Gelatinase-Assapplying (NGAL) and Liver Fatty Acid-Binding Protein (LFABP), have shown promise in early AKI detection [6]. Imaging advancements, such as contrast-enhanced ultrasound (CEUS), provide high diagnostic accuracy for parenchymal lesions, particularly in children and pregnant women. Preventive strategies and early interventions, including aggressive fluid resuscitation, avoidance of nephrotoxic agents, and optimization of hemodynamics, are crucial in mitigating the progression of AKI [7,8]. These approaches play a pivotal role in preventing AKI [9]. By integrating these approaches into existing trauma care frameworks, healthcare providers can enhance the overall quality of care for trauma patients. 

This figure outlines the pathways leading to acute kidney injury (AKI) in trauma patients. Risk factors are divided into “Patient-related” (e.g., comorbidities, fragility, male sex, old age, and hypertension) and “Trauma-related” (e.g., crush injury, brain injury, abdominal injury, hypotension, massive transfusion, and sepsis). The resulting phenotypes of AKI include ischemia (reactive oxygen species/reactive nitrogen species, ROS-RNS), Systemic Inflammatory Response Syndrome (SIRS—damage-associated molecular patterns, DAMPS), sepsis (pathogen-associated molecular patterns, PAMPS), and toxicity (myoglobin). Iatrogenic factors encompass all medical interventions that can potentially become harmful, such as major surgery, resuscitative endovascular balloon occlusion of the aorta (REBOA), hemodynamic alterations, aggressive fluid resuscitation, vasopressor usage, and nephrotoxic agents. The diagram emphasizes the use of biomarkers to identify subclinical AKI. In this condition, there is an increase in biomarkers without clinical AKI, defined by an increase in serum creatinine and/or a decrease in urinary output according to Kidney Disease: Improving Global Outcomes (KDIGO) criteria. The arrows in the diagram illustrate the flow from risk factors through phenotypes and iatrogenic factors, culminating in AKI.

## 2. Epidemiology, Risk Factors, and Predictive Measures

The issue of AKI in trauma patients revolves around the lack of a universally accepted definition, thereby hindering data comparability. The RIFLE criteria (Risk of renal dysfunction, Injury to the kidney, Failure or Loss of kidney function, and End-stage kidney disease) and AKIN definitions established in 2004 and 2007 marked significant milestones [2]. Following these developments, epidemiological studies accumulated, leading to high-quality systematic reviews. A notable systematic review published in 2019 analyzed data from studies conducted between 2004 and 2018 involving more than 25,000 trauma intensive care unit (ICU) patients [2]. The incidence of AKI was found to be 24%, with onset within three days (range 1–6). More than half of these cases presented with mild AKI, and approximately 10% of AKI patients required replacement therapy, constituting 2% of the total patient population [2]. Most patients experienced renal recovery (96%), indicating a high prevalence of elevated renal functional reserve in trauma patients who typically do not have complications from chronic conditions [2].

However, there is no available data regarding long-term chronic kidney disease (CKD) or mortality [2]. Considering the pathophysiology of traumatic kidney injury, the most frequent cause of AKI is related to severe hypotension due to acute hemorrhage [9]. This critical drop in blood pressure reduces renal perfusion, leading to ischemic damage [9]. Organ transplantation serves as a model for understanding ischemia–reperfusion injury [5]. The parallels between ischemia-reperfusion injury in transplanted organs and trauma-induced AKI underscore the importance of studying transplantation-related kidney injury to enhance the approach to mitigating AKI in trauma patients [5]. A high incidence of rhabdomyolysis and elevated serum myoglobin levels predict AKI, particularly with admission values above 1200μg/L or peak myoglobin levels above 5000 μg/L [10,11]. The epidemiological studies provide insights into the typical patient profiles most vulnerable to renal injury, shedding light on the clinical implications of kidney trauma [12,13]. Predictably, factors associated with reduced renal functional reserve feature prominently, including advanced age, pre-existing CKD, diabetes, and hypertension [14]. 

Additionally, the administration of nephrotoxic agents during the treatment of severe trauma patients, such as transfused packed red blood cells, intravenous contrast agents, and hydroxyethyl starch products, significantly contributes to renal stress [15]. Notably, an analysis of odds ratios (ORs) for individual risk factors highlights abdominal trauma and the development of sepsis as significant predictors, surpassing factors like high Injury Severity Scores or shock [16]. In cases of rhabdomyolysis, identifying high-risk patients involves assessing electrolyte imbalances, phosphate levels, creatinine levels, and creatine kinase levels [17]. Furthermore, in severely hemorrhagic patients experiencing low-flow states leading to traumatic cardiac arrest, tests such as NephroCheck^®^, which measure urinary biomarkers of damage [Tissue Inhibitor of Metalloproteinases-2 (TIMP-2) and Insulin-like Growth Factor Binding Protein-7 (IGFBP-7)], can detect and predict AKI within just 3 h of ischemic shock, particularly in trauma cases [18]. Trauma is the central event leading to various complications, such as hemorrhagic shock, which reduces kidney perfusion. Systemic inflammatory response syndrome (SIRS) and rhabdomyolysis release toxic substances that damage the kidneys. The lethal triad of hypothermia, acidosis, and coagulopathy further disrupts normal physiological processes. At the same time, major surgery and the use of resuscitative endovascular balloon occlusion of the aorta (REBOA) can exacerbate the risk of AKI by affecting renal blood flow.

Early identification of such patients allows for proactive measures, such as adjusting blood pressure targets for hypertensive patients, minimizing exposure to nephrotoxic agents, optimizing hemodynamics with advanced monitoring, vasopressor administration, and judicious fluid resuscitation while minimizing the use of colloids [19].

## 3. Renal Diagnostic Approaches in Trauma Patients 

### 3.1. Biomarkers

AKI is traditionally diagnosed through increased serum creatinine (sCr) or decreased urine output (UO), both indicative of reduced glomerular filtration rate and suggesting structural kidney damage [20]. The Kidney Disease: Improving Global Outcomes (KDIGO) criteria define AKI based on an sCr increase of 0.3 mg/dL, 1.5 times the baseline value, or a UO of less than 0.5 mL/kg/h over 6 h [20]. However, these indicators may not fully reflect the actual renal function loss in critically ill patients, where factors such as underlying CKD, medications affecting tubular secretion, and dilution from fluid resuscitation can alter sCr levels [20]. 

Recognizing these limitations, the Acute Disease Quality Improvement (ADQI) group has advocated for including biomarkers in the diagnostic criteria for AKI, integrating these into a new classification system [21]. This refined approach aims to identify “subclinical AKI,” a condition where patients do not exhibit increased sCr yet face similar mortality risks as those with overt AKI [22,23,24]. By incorporating biomarkers, clinicians can detect AKI earlier and potentially intervene before significant damage occurs [25].

The ADQI classification of AKI now includes stages defined not just by traditional measures but also by the presence of specific biomarkers indicative of renal stress or damage [22] (refer to Table 1).

Similar diagnostic advancements have been made in organ transplantation to detect post-ischemic injury early, using biomarkers such as NGAL and LFABP, which have shown promise in early AKI detection in transplanted kidneys [23]. By leveraging these parallels, trauma care can benefit from the diagnostic techniques developed in transplantation.

For instance, biomarkers like NGAL and LFABP have been identified as reliable indicators of structural damage or cellular stress within the kidney [24]. NGAL is particularly noted for its rapid response to kidney injury, appearing in urine and blood within a few hours of damage [6,26]. Similarly, LFABP, released from damaged tubular cells, is a marker of tubular integrity [27]. 

Recent advancements in renal biomarkers have significantly enhanced the early diagnosis and management of AKI, particularly in patients undergoing surgery or those critically injured. Table 2 summarizes the common biomarkers used for the early detection and monitoring of AKI, highlighting their sample types, cutoff values, utilities, and typical applications.

Urinary biomarkers have more complex sample handling requirements compared to blood biomarkers. Hydration status, collection methods, and timing can introduce variability in urinary biomarker results [28,29]. Proper handling and storage conditions are crucial to maintaining urinary biomarkers’ integrity. Immediate processing or specific preservatives may be required to prevent degradation. Additionally, diurnal variations and renal function can influence the concentration of biomarkers in urine, making the standardization of sample collection protocols essential [30]. These complexities highlight the need for rigorous protocols and careful management to ensure reliable urinary biomarker analysis.

Biomarkers, such as NGAL, measured upon admission to the emergency department, have shown high sensitivity and specificity in predicting AKI development. The effectiveness of NGAL as a predictive biomarker is underscored by its high area under the curve (AUC) of 0.869, with a cutoff value of 126.5 ng/mL, making it a reliable indicator of early kidney injury. Another notable innovation in biomarkers for AKI diagnosis is the combination of Tissue Inhibitor of Metalloproteinase-2 (TIMP-2) and Insulin-like Growth Factor Binding Protein 7 (IGFBP7) in urine samples [31]. This biomarker duo indicates cellular stress and signals the need for cell cycle arrest, thereby aiding in the timely initiation of therapeutic interventions. These markers are particularly crucial in identifying patients at elevated risk of severe AKI who may require dialysis following significant injuries. The ongoing research into and clinical application of these biomarkers transforms the approach to AKI management in critical care settings. By enabling earlier detection and more accurate risk stratification, these biomarkers help tailor patient-specific interventions. This proactive approach not only aids in the immediate management of AKI but also plays a pivotal role in preventing its progression to more severe stages or the development of CKD [32]. Moreover, integrating these biomarkers into clinical practice indicates a broader shift toward precision medicine in nephrology. By leveraging these cutting-edge diagnostic tools, healthcare professionals are better equipped to mitigate the renal complications associated with trauma and surgical interventions, improving patient outcomes and care quality in acute and critical settings.

### 3.2. Diagnostic Imaging

#### 3.2.1. Direct Kidney Trauma

Diagnostic imaging, including contrast-enhanced computed tomography (CT), is essential for evaluating renal trauma. CT imaging is the gold standard for assessing renal injuries and renal perfusion in hemodynamically stable patients, as it provides detailed information on the extent and nature of the injury [33,34]. However, CT imaging may not be feasible in hemodynamically unstable patients due to the need to transport the patient to the radiology department, potential contrast-induced nephropathy, and exposure to ionizing radiation.

Several classification systems have been developed to standardize the assessment and management of renal trauma. The World Society of Emergency Surgery (WSES) and the American Association for the Surgery of Trauma (AAST) classifications are widely used to categorize the severity of renal injuries. These systems provide a framework for determining the appropriate therapeutic approach based on the extent of the injury and the patient’s hemodynamic stability (Table 3).

Ultrasound (US) is a critical tool in the early assessment of both renal trauma and AKI due to trauma. The FAST (Focused Assessment with Sonography for Trauma) technique is employed to detect free intra-abdominal fluid, which may indicate severe trauma, including potential kidney injury. This method is particularly valuable for hemodynamically unstable patients where CT imaging is not feasible. A recent monocentric study in pediatric patients with closed abdominal trauma showed how renal ultrasound was effective in detecting severe injuries according to the AAST grades despite being less effective for less severe injuries [35]. For diagnosing AKI due to trauma, renal ultrasound can help identify changes in kidney size, structure, and perfusion. Color Pulsed-Wave Doppler Ultrasound (CPWD-US) is particularly useful in ICU settings for assessing renal perfusion and providing rapid, non-invasive diagnosis of AKI. This technique aids in guiding diagnostic–interventional procedures, such as renal needle biopsy and percutaneous nephrostomy, and monitoring therapy response and AKI progression [8]. CPWD-US in ICU workflows enhances diagnostic precision and patient management despite limited long-term outcome data. 

#### 3.2.2. Trauma-Induced Kidney Injury

Contrast-enhanced ultrasound (CEUS) has emerged as a promising technique for detecting parenchymal lesions and has been suggested to be further investigated, especially in children or pregnant women. CEUS enhances the visualization of renal parenchyma, allowing for detecting subtle injuries that may not be apparent on standard ultrasound [36].

Furthermore, ultrasound can assess renal function and perfusion in trauma patients. By measuring renal blood flow and resistive indices using Doppler ultrasound, clinicians can evaluate the impact of trauma on renal perfusion [37]. Decreased renal blood flow and increased resistive indices may indicate impaired renal perfusion and potential AKI. This non-invasive assessment helps in the early identification of renal dysfunction and guides the management of fluid therapy and vasopressor use in trauma patients [38]. Studies have shown that Doppler ultrasound measurements of renal perfusion correlate with the severity of AKI and can be used to monitor the response to therapeutic interventions. For instance, in trauma patients with suspected renal hypoperfusion, Doppler ultrasound can detect changes in renal blood flow patterns, providing critical information for optimizing hemodynamic support and preventing further renal damage [8,39,40]. While CT scans provide detailed anatomical information, ultrasound offers significant advantages in certain clinical scenarios, particularly in the critical care setting. Ultrasound is readily available at the bedside, does not expose patients to ionizing radiation, and avoids the risks associated with contrast agents. These attributes make ultrasound an invaluable tool for continuously monitoring and assessing renal function and perfusion in trauma patients.

## 4. Strategies for Mitigating AKI Impact in Trauma Management

Modern trauma management, including permissive hypotension, early use of blood products, and damage control surgery, has improved prehospital and in-hospital mortality [41,42]. Hypotensive resuscitation has shown benefits on mortality, and a recent meta-analysis reported no increase in the incidence of AKI when this strategy was applied [43]. However, there are limited data on blood pressure targets beyond 24 h post-trauma. Clinicians generally adhere to recommended perfusion parameters, often maintaining a mean arterial pressure (MAP) of at least 65 mmHg. The following approaches are critical for mitigating the impact of AKI in trauma management: (1) Volume and Choice of Fluid Therapy; (2) Restriction of Crystalloid Therapy; (3) Potential Benefits of Colloids; (4) Hemoglobin Management; and (5) Use of Vasopressors.

Volume and choice of fluid therapy are crucial in the management of trauma patients during both early resuscitation and later stages. Fluids are categorized into crystalloids and colloids. Colloids contain oncotic macromolecules that largely remain in the intravascular space, whereas crystalloids, composed of smaller molecules and electrolytes, cause volume expansion via sodium but lack oncotic properties. This distinction suggests colloids should theoretically require less volume for fluid expansion, though this has not been meaningfully demonstrated in practice [44]. 

Modern trauma management restricts *crystalloid* therapy in prehospital and acute resuscitation settings [45]. Increased mortality has been consistently observed with more liberal use of crystalloids during the first 24–48 h of admission in both adult [46] and pediatric trauma patients [47]. When fluid is required, crystalloids remain a common choice both prehospital and during critical care admission. Compared to isotonic saline (0.9% salt solution), balanced solutions are considered safer, especially when larger volumes are needed [48,49]. Excessive chloride administration from isotonic saline can lead to hyperchloremic metabolic acidosis, which has been associated with renal vasoconstriction, decreased renal blood flow, and an increased risk of AKI and mortality [50,51]. A retrospective study found an association between hyperchloremia (chloremia higher than 110 mmol/L) and mortality in trauma patients [52]. This relationship highlights the importance of fluid composition in influencing renal outcomes in trauma patients. Balanced crystalloids, which contain lower chloride concentrations, help maintain acid-base balance and reduce the risk of hyperchloremia, thus potentially decreasing the incidence of AKI.

The potential benefit of colloids lies in their ability to induce a more rapid and persistent plasma expansion due to increased oncotic pressure. The CRISTAL study compared colloids (including gelatins) to crystalloids in hypovolemic shock but found no differences in 28-day mortality [53]. Thus, crystalloids should remain the first-line therapy to correct hypovolemia in hemorrhagic shock. Hemoglobin, the oxygen transporter, is critical in delivering oxygen to renal tubules. Experimental work suggests that correcting anemia with transfusion normalizes cortical and medullary PO2 during hemorrhage resuscitation, whereas fluid resuscitation alone does not improve renal PO2 [54]. A study involving 965 cardiac surgery patients found that AKI was associated with low hemoglobin levels during surgery and postoperative care [55]. Vasopressors are typically used to increase blood pressure when fluid resuscitation fails to meet mean arterial pressure goals.

Norepinephrine is the first-choice drug in many shock cases, with vasopressin as a secondary option per the Surviving Sepsis guidelines [56]. Some studies suggest vasopressin’s potential superiority in kidney outcomes compared to norepinephrine. The VASST trial found no difference in kidney dysfunction or need for renal replacement therapy (RRT) between norepinephrine and vasopressin [57]. However, a posthoc analysis using the RIFLE criteria found that vasopressin was associated with a lower likelihood of progressing to more severe renal categories and lower RRT rates than norepinephrine [58]. The VANISH trial showed that the early addition of vasopressin to norepinephrine was associated with higher urine volumes and lower RRT rates in septic shock patients [59]. High-dose norepinephrine can decrease kidney and mesenteric blood flow, while vasopressin has minimal vasoconstrictive effects on renal afferent arterioles, improving kidney blood flow [60,61]. Despite potential adverse effects like digital ischemia, vasopressin may be preferred for patients at risk of AKI [62].

In conclusion, the high incidence of AKI in trauma patients necessitates early identification of those at risk to establish a resuscitation strategy aimed at preventing AKI. The choice and composition of resuscitation fluids play a significant role in this strategy, as they can influence renal perfusion and function. Balanced crystalloids, appropriate use of colloids, and careful management of hemoglobin levels and vasopressors are critical components in mitigating the risk of AKI. By understanding these relationships, clinicians can better tailor their resuscitation approaches to improve outcomes for trauma patients.

## 5. Holistic Trauma Care Dynamics

Holistic trauma care dynamics represent an integrated approach to trauma management that emphasizes comprehensive patient care beyond immediate injury stabilization [63]. This concept recognizes the complex medical needs of trauma patients, addressing both acute and long-term health issues through a multidisciplinary, collaborative approach. Two key components of this holistic strategy are team-based precision and the integration of AKI management into comprehensive trauma protocols.

### 5.1. Team-Based Precision: A Multidisciplinary Approach to Trauma Care

Traditionally, trauma care has been compartmentalized, with different specialties focusing on specific aspects of patient management [64]. However, contemporary trauma care emphasizes a multidisciplinary team-based approach that leverages the expertise of various healthcare professionals to provide comprehensive and individualized care. Collaboration among trauma surgeons, emergency physicians, intensivists, nurses, pharmacists, respiratory therapists, and other allied health professionals Field [64] is at the core of team-based precision. Each member brings unique skills and perspectives, facilitating comprehensive assessment, treatment, and ongoing monitoring of trauma patients. The multidisciplinary team model enables rapid assessment and intervention, optimizes resource utilization, and improves patient outcomes. By leveraging the collective expertise of diverse healthcare professionals, trauma centers can deliver personalized care tailored to each patient’s specific needs.

### 5.2. Infusing AKI Management into Comprehensive Trauma Protocols

AKI is a common and potentially devastating complication in trauma patients, contributing to increased morbidity and mortality [1]. Despite its significance, AKI management has traditionally been addressed in a fragmented manner within trauma protocols. Integrating AKI management into comprehensive trauma protocols involves several key steps:

- Standardized Protocols: Implementing standardized protocols for AKI risk assessment, early detection, and management. This includes routine monitoring of renal function markers, such as serum creatinine and urine output, coupled with risk stratification based on injury severity and comorbidities [43];
- Preventive Strategies: Prioritizing strategies to prevent AKI, including aggressive fluid resuscitation, avoidance of nephrotoxic agents, optimization of hemodynamics, and early recognition and treatment of conditions predisposing to renal injury, such as rhabdomyolysis and hypovolemia [45,65];
- Evidence-Based Interventions: When indicated, incorporate evidence-based interventions for managing established AKI, including renal replacement therapy. Timely initiation of appropriate interventions can mitigate the progression of AKI, improve renal recovery, and ultimately enhance patient outcomes [53,54];
- Education and Quality Improvement: Ensuring adherence to AKI management protocols through ongoing education and quality improvement initiatives. This includes regular training sessions, performance feedback, and continuous evaluation of protocol effectiveness and adherence [66].

By infusing AKI management into comprehensive trauma protocols, healthcare institutions can enhance the quality and consistency of care provided to trauma patients, reduce the incidence and severity of AKI-related complications, and improve overall clinical outcomes. Holistic trauma care dynamics encompass a multidisciplinary approach to patient management that addresses the diverse medical needs of trauma patients. By embracing team-based precision and integrating AKI management into comprehensive trauma protocols, healthcare institutions can optimize patient outcomes and deliver high-quality care across the continuum of trauma care.

## 6. Frontiers in Trauma Research

The frontier of trauma research is rapidly advancing, propelled by a deeper understanding of the biological and molecular underpinnings of AKI [6]. Innovations in genomic and proteomic technologies are unlocking opportunities to dissect the pathways triggered by traumatic events leading to kidney injury. This exploration is set to reveal new biomarkers and therapeutic targets that could revolutionize the prediction, diagnosis, and management of AKI in trauma patients. The focus is identifying early predictive markers that can be monitored in real-time, allowing for pre-emptive therapeutic interventions. Looking ahead, the therapeutic landscape for AKI is on the brink of transformation. Advances in regenerative medicine, including stem cell therapy and organ bioengineering, hold promise for repairing kidney damage at the cellular level [67].

Developing novel pharmacological agents to mitigate oxidative stress, inflammation, and cellular apoptosis also offers hope for enhancing renal recovery. Despite these advancements, prevention remains the cornerstone of managing trauma-associated nephropathy [68,69]. Enhanced protocols for fluid management, refined strategies to avoid nephrotoxic agents, and improved hemodynamic monitoring form an evolving preventive paradigm that prioritizes renal preservation as a fundamental aspect of trauma care [70]. This integrated approach underscores the proactive shift toward minimizing the impact of renal injury following trauma, aiming to improve patient outcomes through both innovative treatment and rigorous preventive measures.

## 7. Conclusions

Incorporating biomarkers into the diagnostic criteria for AKI represents a significant advancement in managing renal complications, particularly in critically ill patients. Traditional methods relying solely on serum creatinine and urine output are often insufficient, especially in complex clinical scenarios involving trauma and surgical interventions. Biomarkers such as NGAL, LFABP, and the combination of TIMP-2 and IGFBP7 provide a more sensitive and specific means of detecting renal stress and damage early. This proactive approach enables clinicians to initiate appropriate interventions sooner, potentially mitigating the progression of AKI and improving patient outcomes. The ongoing research and clinical application of these biomarkers signify a broader movement toward precision medicine in nephrology, offering a promising avenue for enhancing the quality of care in acute and critical settings.

## Figures and Tables

**Table 1:** ADQI classification of acute kidney injury based on functional criteria and biomarker results.

Stage	Criteria	Biomarker Results
**1s**	No change in sCr or UO	Positive
**1A**	Increased sCr ≥ 0.3mg/dL within 48 h or ≥1.5 times basal value and/or UO < 0.5 mL/kg/h for 6 h	Negative
**1B**	Same as 1A	Positive
**2A**	Increased sCr ≥ 2 times basal value and/or UO < 0.5 mL/kg/h for 12 h	Negative
**2B**	Same as 2A	Positive
**3A**	Increased sCr ≥ 3 times basal value and/or UO < 0.3 mL/kg/h for 24 h or anuria for >12 h or RRT requirement	Negative
**3B**	Same as 3A	Positive

**Table 2:** Summary of the common biomarkers used for the early detection and monitoring of AKI.

Biomarker	Sample Type	Cutoff Value	Utility	Typical Application	
NGAL	Plasma or Urine	150 ng/mL	Predicts AKI; sensitive and specific	Used upon admission in emergency settings for trauma patients	Plasma or Urine
LFABP	Urine	50 ng/mL	Indicates tubular damage	Used in critical care to detect early structural kidney damage	Urine
IL-18	Plasma or Urine	200 pg/mL	Associated with inflammatory response in AKI	Used to differentiate between various causes of AKI	Plasma or Urine
TIMP-2	Urine	TIMP-2 × IGFBP7 > 0.3 (AKIRisk™)	Indicator of cellular stress	Part of a combination test with IGFBP7 for early AKI detection in ICU patients	Urine
IGFBP7	Urine	TIMP-2 × IGFBP7 > 0.3 (AKIRisk™)	Indicates cell cycle arrest	Combined with TIMP-2 to assess risk of AKI development post-surgery	Urine

**Table 3:** Innovative comparison of renal trauma classification systems: WSES vs. AAST.

WSES (2018 Revised)	AAST	Detailed AAST Description	Hemodynamic Stability
I	1	Subcapsular hematoma and/or parenchymal contusion without laceration	Stable
	2	Perirenal hematoma confined to Gerota fascia	
		Renal parenchymal laceration ≤1 cm depth without urinary extravasation	
II	3	Renal parenchymal laceration >1 cm depth without collecting system rupture or urinary extravasation	
		Any injury with kidney vascular injury or active bleeding contained within Gerota fascia	
III	4	Parenchymal laceration extending into urinary collecting system with urinary extravasation	
		Renal pelvis laceration, complete ureteropelvic disruption, or both	
		Active bleeding beyond Gerota fascia into retroperitoneum or peritoneum	
		Segmental renal vein or artery injury or kidney infarction(s) caused by vessel thrombosis without active bleeding	
	5	Shattered kidney with loss of identifiable parenchymal renal anatomy	
		Devascularized kidney with active bleeding	
		Main renal artery or vein laceration or avulsion of hilum	
IV	Any	Any	Unstable
